# Implementing measurement-based care in virtual mental health services for rural veterans: provider insights from a pre-implementation evaluation

**DOI:** 10.1186/s12913-026-14490-6

**Published:** 2026-04-03

**Authors:** Amanda Heeren, Kimberly McCoy, Lindsey Fuhrmeister, Natalie Suiter, Jan A. Lindsay, Chloe Hoang, Laura Marsh, John Fortney, Carolyn Turvey

**Affiliations:** 1 Iowa City Veterans Affairs Health Care System, Veterans Rural Health Resource Center-Iowa City, VA Office of Rural Health, 601 Highway 6 W, Iowa City, IA 52246 USA; 2 Department of Psychiatry, Carver College of Medicine, University of Iowa, 200 Hawkins Drive, Iowa City, IA 52242 USA; 3 School of Social Work, University of Iowa, 20 W Davenport St, 308 NH, Iowa City, IA 52242 USA; 4 Michael E. DeBakey VA Medical Center, VA HSR&D Houston Center of Innovations in Quality, Effectiveness and Safety, 2002 Holcombe Blvd, Houston, TX 77030 USA; 5 Department of Medicine, Baylor College of Medicine, 1 Baylor Plaza, Houston, TX 77030 USA; 6 Education, and Clinical Center, South Central Mental Illness Research, 2002 Holcombe Blvd, Houston, TX 77030 USA; 7 Michael E. DeBakey VA Medical Center, Mental Health Care Line - Behavioral Health Interdisciplinary Program, 2002 Holcombe Blvd, Houston, TX 77030 USA; 8 Departments of Psychiatry and Neurology, Baylor College of Medicine, 1 Baylor Plaza, Houston, TX 77030 USA; 9 Department of Psychiatry and Behavioral Sciences, School of Medicine, University of Washington, 1959 NE Pacific St, Seattle, WA 98195 USA; 10 VA Health Systems Research, Center of Innovation for Veteran-Centered and Value-Driven Care, VA Puget Sound, 1660 S Columbian Way, Seattle, WA 98108 USA

**Keywords:** Measurement-based care, Veterans health administration, Veteran mental health, Rural healthcare, Tele-mental health, Digital workflow, Quality improvement, Telehealth, Digital mental health, Provider training

## Abstract

**Background:**

Measurement-Based Care (MBC) uses repeated standardized psychological assessments to monitor treatment progress and guide clinical decision-making. Although MBC improves outcomes when results are discussed collaboratively, adoption across the Veterans Health Administration (VHA) remains below 50%, particularly in virtual and rural settings. This pre-implementation evaluation explored provider perspectives to inform development of a digital MBC workflow for rural tele-mental health settings.

**Methods:**

Using the Alacrity Center’s Discover, Design, Build, and Test model and User Centered Design, we conducted a pre-implementation survey of mental health providers at a VHA Medical Center providing care to rural and highly rural Veterans in South Central United States. Providers represented disciplines of psychology and social work. The survey contained structured and open-ended questions assessing prior MBC use, perceived benefits and drawbacks, and educational needs. Qualitative data underwent inductive thematic analysis by two independent coders using consensus review to identify emergent themes.

**Results:**

Forty-six providers identified three key benefits of MBC: enhanced symptom awareness and monitoring, support for data-informed treatment planning, and facilitation of patient engagement and shared decision-making. Reported barriers included time constraints, inconsistent Veteran participation, functionality of digital tools, and challenges in interpreting and integrating results. Providers also expressed concern over the accuracy of self-report measures and potential negative psychological effects of repeated symptom tracking. Educational needs clustered around three domains: (1) psychometric understanding of measures, (2) effective communication of results with Veterans, and (3) practical guidance on integrating digital tools into workflow. Illustrative quotes are presented verbatim.

**Conclusions:**

Provider feedback underscores that successful MBC implementation in digital mental health requires not only technological infrastructure but also targeted provider education and psychoeducational tools. Training should emphasize interpretation of assessment data, patient-centered communication, and safe, ethical management of sensitive results. Embedding MBC within a structured digital workflow supported by provider-informed design can enhance engagement, streamline care, and align with VA’s goal to expand high-quality, data-driven mental health services for rural Veterans. Given the quality improvement context, findings should be interpreted as context-specific insights used to shape local implementation rather than generalizable evidence.

**Supplementary Information:**

The online version contains supplementary material available at 10.1186/s12913-026-14490-6.

## Background

Effective mental health treatment increasingly emphasizes ongoing evaluation and data-driven decision-making approaches, such as measurement-based care (MBC), to guide clinical practice and improve outcomes. MBC in mental healthcare entails the repeated administration of standardized psychological and psychiatric symptom assessment measures completed by patients to monitor treatment response and inform subsequent care decisions. MBC, a cornerstone of evidence-based care, contributes to learning health system approaches when its data are aggregated at the clinic or network level [1, 2]. However, the potential of MBC cannot be realized if it is not implemented effectively or provider adoption is low. It is estimated that less than 20% of mental health providers nationwide use MBC [3–5].

Considerable evidence indicates that MBC only improves clinical outcomes when both the patient and provider receive results and openly discuss them to inform treatment decisions [6–10]. To date, healthcare systems implementing MBC at scale struggle to meet this best practice [11]. In a 2015 meta review by Krageloh et al., findings underscore the importance of providers reviewing results collaboratively with patients for MBC to have clinical impact [10]. However, even when systematic collection of psychological measures and reporting of results to patients and providers occurs, there is frequently a failure to integrate results into care discussions.

The Veterans Health Administration (VHA), the largest healthcare system in the United States, has a national office promoting MBC to improve services for Veterans receiving mental health care. VHA’s model uses a “Collect, Share, Act” framework [12], emphasizing that coordination of all three key components is required to achieve better treatment outcomes. “Collect” includes introducing MBC to the patient, selecting measures and explaining to patients why those measures were selected, and regularly and repeatedly administering the measures. The “Share” component emphasizes reporting and discussing the scores with the patients, verifying that the scores reflect the patient’s subjective experience, and capturing the data in the medical record. “Act” refers to the processes of the clinician identifying if data trajectory indicates improvement, worsening, or no change, the clinician and patient collaboratively exploring ideas for treatment, and jointly deciding on treatment changes.

Even with nationwide efforts by VHA to support MBC use, widespread provider adoption of telehealth-delivered MBC (tMBC) within VHA has not yet been achieved. A 2019 VHA study found only 15% of patients had MBC assessments despite a concerted use of implementation strategies by VA’s Office of Mental Health [13] that have been found to increase MBC implementation rates.

Challenges to tMBC adoption in rural VHA Community-based Outpatient Clinic (CBOC) settings extend beyond the challenges faced in adoption of MBC delivered in person. VHA utilizes VA Video Connect (VVC) as its video-based telehealth platform. VHA has aimed to support providers by developing mobile health applications, including decision support software Behavioral Health Lab Touch^®^ (BHL Touch) [14, 15], eScreening [16], and Mental Health Assistant [17] to transition the collection, analysis, and sharing symptom trajectories with patients from paper and pencil to an electronic platform. This shift aligns with civilian practices, with numerous digital platforms being developed and implemented to support tMBC [18, 19]. However, inconsistent implementation, provider education, workflow integration, and tool utilization persist [20]. The two main usability barriers are inadequate informatics support and workload/time burden [13, 21–23].

VHA CBOC facilities often lack adequate staff or informatics support to facilitate a tMBC workflow in virtual care delivery and as a result opportunities for optimal care are limited by low rates of MBC adoption by tele-mental health providers for rural Veterans. Historically, utilizing MBC during VHA telemedicine encounters was complicated for both providers and patients. Administration of MBC assessments required verbal administration of the tool by the clinician or sending physical copies of assessments to a Veteran prior to the appointments. For the latter, the patient could then hold the completed paper version of the assessment up to the camera or return it by mail to the providers, who would enter the responses in the VHA patient-reported outcome measures applications such as BHL Touch or Mental health Assistant.

Our quality improvement (QI) project *“Virtual Care QUERI Program: Implementation of Technology Facilitated Evidence Based Practices to Improve Access to High Quality Care for Rural Veterans*” (project number QUE 20 − 007) was funded by VA’s Quality Enhancement Research Initiative, a national program that partners with VHA providers, leaders, and Veterans to broaden reach and adoption of evidence-based practices across the United States [24]. The project emphasizes Krageloh et al.’s mandate that MBC go beyond the collection of questionnaires to a process in which results are actively shared and discussed [10]. When patients complete these measures, providers often do not review the results and rarely discuss them with patients.

The aim of this project was to improve rural Veterans’ access to MBC by optimizing digital delivery and adapting workflows for tele-mental health providers while maintaining the three core components as specified in existing VHA MBC Implementation Guidelines: (1) COLLECT repeated assessments over time, (2) SHARE results between patient and provider during the encounter, and (3) ACT upon data in treatment decisions after reviewing patient trajectories over time.

Designed prior to the COVID-19 pandemic, this QI project sought to collaborate with VHA Mental Health Service Lines, comprised of a VAMC and its affiliated CBOCs, to establish a feasible, efficient, and secure digital workflow for patient assessment and results review when using telehealth. The prospect of an efficient digital workflow process revealed the valuable role of digital health innovations in extending evidence-based mental health services to Veterans in geographically remote areas. As project implementation began during the COVID-19 pandemic, when in-person meetings and direct patient care were significantly limited, the project’s timeliness and salience to VHA tele-mental health care, in general, was serendipitous.

This QI project developed and refined an intervention to improve uptake of MBC among VA psychotherapy providers. This pre-implementation evaluation explored provider perspectives on barriers, facilitators, and educational needs related to adoption of tMBC in virtual mental healthcare delivery. This manuscript reports on qualitative findings from a pre-implementation survey conducted during the Discover phase and how results informed subsequent stages of the project.

## Methods

### Study design

Guided by the ALACRITY Center’s Discover, Design, Build and Test model [25] and User Centered Design [26, 27], the project initially proposed to conduct in-person site visits to complete qualitative interviews and direct observations of clinician routines to map clinical workflows. However, in response to evolving clinic-wide practices during the COVID-19 pandemic, the project was adapted by leveraging the virtual platform Microsoft Teams to allow the project team to attend staff meetings (held virtually) at participating sites. These monthly virtual meetings with clinic teams became the primary venue for implementation and training activities.

The initial project meeting with participating psychotherapy providers occurred during the service line’s standing staff meeting. During this initial meeting, the QI team oriented providers to the project and invited staff in attendance at the meeting to complete a baseline assessment of their perceptions of and experiences with MBC.

Subsequent monthly meetings included a variety of feedback loops and educational topics. Feedback loops comprised a presentation of survey results, workflow redesign discussions using “think aloud” protocols to determine how to breakdown MBC sub-components and troubleshooting key barriers. Educational components included overviews of the VHA’s “Collect, Share, Act” framework, case presentations, available assessments, and the BHL Touch technology for electronic assessment capture. Within VA, the most commonly used patient reported outcome measures are the Patient Health Questionnaire-9, Generalized Anxiety Disorder-7, and PTSD Checklist for DSM-5. In addition to educating providers on those measures, we emphasized the importance of selecting measures aligned with patient-identified treatment goals, such as those assessing functioning and wellbeing. In fact, the dissemination of a comprehensive assessment library was to support clinicians in choosing measures that best reflect these individualized goals. Although VHA offers multiple software options to support tMBC, project leadership believed training in multiple available technologies would overcomplicate a process we were intending to simplify. As such, BHL Touch was the primary software targeted in the QI project. To address known barriers to adoption [13, 21], we expanded upon VHA’s MBC Implementation Guide by more fully promoting electronic data capture to reduce workflow burden. At the end of participation, a final meeting featured a wrap-up to consolidate learning and plan for maintenance.

Participating providers were instructed to use their clinical judgment to establish sustainable workflows related to assessment selection, frequency, and delivery time of assessments based on current and preferred workflows. As BHL Touch was the primary platform being emphasized in this project, providers could select from the library of assessments available in BHL Touch, or in some cases, they could send assessments they deemed clinically appropriate that were not in the BHL Touch library. Frequency of measurement was also left to the discretion of the provider to accommodate clinical judgement and Veteran preference. Some providers elected to send the assessments at the beginning of each week as a part of their overall workflow. Others elected to send them as close as 15 min prior to the schedule appointment. As part of the project, providers were educated on how to use BHL Touch’s automated process to schedule and send assessments.

Project participation lasted 6 months. This structured approach allowed the program to be flexible yet consistent by embedding MBC within digital health infrastructures to support VHA’s mission to expand high-quality, data-driven care, particularly for rural and underserved Veterans.

This QI project was determined to be non-human subjects research by the University of Iowa Institutional Review Board. Per VHA Handbook 1200.21, all VA authors of this manuscript attest that the activities that resulted in producing this manuscript were not conducted as part of a research project, but as part of a non-research evaluation conducted under the authority of the Office of Mental Health.

### Participants

Participants were psychotherapy providers including psychologists and clinical social workers from a VA Medical Center Mental Health Service Line serving rural and highly rural Veterans in the South Central US. The service line was identified and recruited by the project lead (CT) due to the CBOCs’ proportion of rural service areas.

Participation in the MBC project was not mandatory; however, the baseline evaluation and subsequent project training sessions were held during the service line’s regularly scheduled staff meetings. All therapists in the clinic receive MBC training as part of their clinic orientation. Re-trainings occur yearly at a minimum. The implementation of MBC is written in the therapists’ performance plan and ongoing peer professional evaluation, which occur quarterly.

### Pre-implementation survey

As part of the Discover phase, we conducted a pre-implementation survey with structured and open-ended questions. Data was collected in August 2023. An electronic survey link, distributed via Qualtrics, was sent to the 57 providers in attendance at the initial project meeting, which occurred during a regularly scheduled staff meeting. The total number of psychotherapy providers within the service line was variable throughout the duration of the project, due to staff turnover, medical leave, and job reassignment. Staff were informed that the survey was optional, responses were anonymous, and that they could skip any question they did not want to answer. Consent was implied via survey participation. The full survey is available as Appendix 1. Qualitative and demographic data collected from the survey are reported here.

Demographic data were collected via closed ended questions. Qualitative data collected in the survey assessed provider opinion on benefits and drawbacks of MBC and education needs related to adding MBC to the provider’s clinical toolbox. Survey items were developed *de novo* by the multidisciplinary project team to align with the objectives of the pre-implementation evaluation. The survey was collaboratively refined by team members representing clinical (CT, AH, MR) and implementation science (AH, LF) expertise to ensure relevance and face validity within the VHA context. Consistent with the rapid and iterative nature of QI work, the survey was not pilot tested prior to administration.

### Analysis

Data obtained through Qualtrics was cleaned, managed, and analyzed using the Statistical Analysis System 9.4 (SAS) [28]. Descriptive statistics, including frequencies and summary measures, were used to analyze the demographic data of participating providers. All data were de-identified prior to sharing, and VHA secure data storage protocols were followed.

Descriptive statistics were used to analyze the demographic data of participating providers. Data from clinical social workers and psychologists were aggregated to support development of an implementation model that met the needs of the psychotherapy providers within the Mental Health Service Line. Inductive thematic analysis [29, 30] was applied by two members of the project team (AH, CT) to the qualitative data. Emergent themes were identified through the development of a codebook. To develop the codebooks, AH and CT together systematically reviewed 5 responses from each question, discussed key concepts, and agreed upon codes. The remaining responses were coded individually by CT and AH, iteratively developing the codebook until no new codes were identified and saturation was reached. A team meeting was held to achieve consensus when clarification was needed to address or revise codes.

Data analyzed for this manuscript was utilized in an iterative feedback loop supporting the ongoing QI project. The timely analysis of this data was important to inform the Design phase.

This manuscript was developed in accordance with the Standards for Quality Improvement Reporting Excellence guidelines [31] (Appendix 2).

## Results

A total of 46 psychotherapy providers participated in the electronic survey, including psychologists (*n* = 28), licensed clinical social workers (*n* = 17), and a non-licensed social worker (*n* = 1). Of the participants who shared information on their age, 37% (*n* = 17) were aged 25–39, 54% (*n* = 25) were aged 40–59, and 2% (*n* = 1) were 60+. Survey participants were asked, “For what percentage of your patients do you employ measurement‑based care?” Among the 40 providers who indicated that they use measurement‑based care with Veterans in their clinical practice, reported usage with patients ranged from 9% to 100%, with a mean of 79.45% and a median of 82%.

Results presented here are organized by perceived benefits, drawbacks, and educational needs related to MBC. The identified sub-themes provide insight into the nuances of implementing MBC and integration of the practice into clinical workflows. Quotes are reported verbatim (written).

### Benefits

Many benefits of MBC were reported by participating providers and are organized across three subthemes: symptom awareness and monitoring, guiding clinical decision making, and facilitation of care delivery.

#### Symptom awareness and monitoring

Twenty-six participants (56.5% of the total respondents) reported the benefit of symptom awareness and monitoring, including identification of symptoms, illumination of severity of symptoms, and the ability to easily track symptoms over time. Eleven participants (23.9% of total respondents) reported that the assessments help identify symptoms that were not revealed during clinical interactions, with one provider sharing, “[MBC] *Helps to guide the treatment and open a forum for discussing specific symptoms that may otherwise go unnoticed”* [ID007]. Another provider explained, *“It helps determine what symptoms may be more prevalent in a patient’s situation. The Veterans are usually not aware of the degree of their depression for instance until they complete the PHQ-9”* [ID0036]. Another noted, “*[MBC] helps you to be thorough and not miss symptoms in your assessment of current concerns”* [ID0040].

Insight into symptom severity can be helpful in ongoing treatment planning, and as noted in the survey responses, Veterans may not be aware of the severity of their symptoms. One provider spoke to the benefit of quantifying symptom severity, expressing that MBC *“Puts symptom severity into an easier to understand number”* [ID0028].

One provider summed up the benefits of symptom awareness and the subsequent opportunity for guidance in treatment decision-making, stating, *“Significant benefits are diagnostic clarity*,* current snap shot of ‘mental coordinates’*,* opportunity to discuss current factors*,* see improvement*,* make adjustments*,* understand their thought process and why they answer the way they did*,* provides feedback and other data such as habit and automatic thought process in responses if there are not any changes”* [ID0035].

#### Guiding clinical decision making

MBC can guide clinical decision-making through objective tracking of symptom change (or lack thereof), identifying progress toward care goals, and informing treatment and, ultimately, discharge planning.

Providers noted benefits of collecting assessment data, indicating that when first administered the assessment results give a baseline from which the response to treatment can be monitored. One provider shared, “*It allows you to better understand the patient and gives you a baseline at which to measure the efficacy of treatment*” [ID0041]. As data collection continues over the course of treatment, progress can be measured and viewed in a more objective manner. According to one provider, *“It helps provide additional data to patient and clinician’s subjective experience and judgement to determine the effectiveness in treatment”* [ID0033]. This objective understanding of the symptom change was noted as important by participating providers.

Providers spoke about how MBC’s provision of insights into nuanced symptom changes is valuable for treatment planning by helping to refocus goals and priorities and support patients’ ability to identify and act on areas of concern. According to one provider, MBC *“provides a structure for involving the patient in a discussion regarding progress (or lack of)”* [ID0002]. Other providers spoke about how MBC is good for identifying unmet needs, noting MBC helps with “*tracking progress relative to goals [and to] identify sources of support needed that may not be being addressed by the current therapy”* [ID0009], and *“For me and the patients to track their progress over time and see when therapy is making a difference and if not*,* where they may be stuck”* [ID0010].

Respondents reported how the psychoeducation component of MBC is a valuable tool in helping patients to better understand their diagnoses and symptom severity. *“Given the need for episodic care and the frequency with which Veterans present with complex*,* chronic problems*,* I think use of MBC can help both Veteran and provider feel clear and satisfied about progress in treatment. I work almost exclusively with patients with chronic issues because of my specialty and use of measures helps educate patients that fluctuations in symptoms do occur and help them think about quality of life beyond symptom focus”* [ID0039].

Outside of the psychotherapy realm, one provider spoke about MBC’s value in psychotropic medication management. *“For Veterans that are taking medications, if working, then I want to see their symptoms to be moderate to mild vs. moderate to severe. Then it can indicate a need to review medications to either increase dosage, maintain the dosage or even lower dosages at times”* [ID0036].

#### Facilitation of care delivery

Providers conveyed that MBC facilitates engaging Veterans in treatment, supports rapport building, saves time and helps Veterans move toward acceptance of a clinical diagnosis. Helping Veterans feel more engaged in their own treatment was noted to be a benefit among many providers. Providers articulated important insights into their perceived value of MBC saying, *“completing the assessments during session strengthens partnership”* [ID043], that MBC “*… helps patients feel more involved in their own care”* [ID0004], “*validates a Veteran’s experience*” [ID0011], “*[provides] better education about mental health conditions”* [ID0012], and “*can help people improve their self-monitoring and self-awareness of different symptoms and make links between symptoms and an overall diagnosis.”* [ID0038].

Time was discussed in the report of benefits of MBC. MBC was reported as being a time-saver by some, but not all providers. One provider shared *“The biggest benefits of MBC are its use during intake assessments (almost as a ’short cut’ during these long assessments to help with time management)*” [ID0006]. Time was also discussed in the context of being able to identify problem areas and make change right away, “*Being able to track progress and act on areas of concern right away is invaluable. This is one of the other major pros of MBC*” [ID0019].

In addition to the clinical benefit of MBC, some providers described adjunctive benefits, specifically how MBC processes can act as a facilitator of care. One beneficial factor associated with successful uptake of clinical interventions is the ease with which individual technological systems (i.e. BHL Touch and VHA’s electronic health record (EHR)) work cohesively in support of the intervention. Some providers discussed the ease with which MBC can be integrated into video telehealth visits with one provider indicating that BHL touch provides an advantage in MBC, stating “*Since I started using BHL touch*,* I no longer feel I have a disadvantage to face-to-face providers (who can hand a form to a client*,* have them fill it out*,* then look it over later)”* [ID0006]. Providers also pointed out the added benefit of the text message sent to collect symptom data acting as a secondary appointment reminder.

### Drawbacks

Providers’ reports on the drawbacks of MBC spanned three categories: assessment completion and self-report bias, structural barriers to provider engagement, and obstacles to evidence-based practice model adherence.

#### Assessment completion and self-report bias

Providers reported a drawback of MBC is that Veterans do not always complete the assessments, noting the circumstances in which this happens and a variety of possible reasons. Although reasons for non-participation are unique to each Veteran, providers cited a variety of perspectives as to why some Veterans may not complete assessments, including MBC not aligning with a Veteran’s goals, provider concerns about MBC having a negative impact on a Veteran, the possibility of self-report bias or response distortion, discouragement from stagnant scores or negative trends, and fear of impact on VA disability benefits. Incomplete assessments were reported to be expressly difficult in digital settings, with one provider describing how, *“when using during a telehealth visit- it can be hard to get compliance when asking them to do it prior to the session”* [ID0026].

Providers noted that perhaps Veterans are inconsistent in completing assessments due to the Veteran’s perspective on the value of MBC. Some providers reported that the number of instruments used and the frequency of assessment places a burden on some Veterans, leading to MBC feeling “*clunky*” and *“less personalized for patient care*”. One provider shared *“Patients won’t always respond or get frustrated with doing them.”* [ID0046]. The value of MBC is not always realized immediately among Veteran patients, making it difficult to get *“Veteran buy in”* to see the benefits.

Concerns about negative psychological impacts on Veteran patients were communicated.

The psychological impacts described included frustration, demoralization, discouragement, and anxiety provocation. One provider shared their experience that *“For some Veterans*,* it reinforces how poorly they are doing to fill the questions out week to week…and it raises anxiety to fill out the questionnaire about how their anxiety is. There are cases where the measurement process really bothers Veterans. In those cases*,* I make adjustments rather than disqualify them from an evidence-based practice (assuming there are not other issues such as non-compliance from homework or high resistance to the structured session format)”* [ID0006]. Further speculation on how MBC may impact the Veteran was conveyed by one respondent saying *“Some people communicate that seeing the numbers can elicit shame or discouragement that they are not improving*,* even though people in therapy tend to get worse before they get better”* [ID0038].

Providers conveyed apprehension in accuracy of reported symptom data, due to self-reporting bias and reporting distortion. In some cases, inaccurate symptom data may be due to questions not being specific enough or fatigue in filling out the assessments. One provider expressed how MBC can create difficulties in the session, noting that issues arise “*When Veterans want to add to the questions because they are not specific enough or when Veterans elaborate too much on each question or get frustrated by questions (especially in longer assessments*,* like BASIS 24 and IMRS”* [ID0022].

Some providers posited that response distortion may be influenced by a desire to seem better than they are or by a fear of reduction in benefits due to improvement. In these cases, providers indicated reticence to use MBC. Regarding reporting that may minimize appearance of symptom burden, one provider stated, “*Some Veterans ‘fake good’ and engage in more denial and minimization of symptom*,* so the numbers are reflective of what they report in sessions.”* [ID0038].

Further concern about self-report bias and response distortion was raised around the issue of suicidality. *“Patients learning how to respond to the measure to avoid having difficult conversations or hospitalization. For example, a Veteran might learn that if they endorse having suicidal thoughts, they will get follow up questions that might lead to being hospitalized. Rather than saying, ‘I need to have this conversation,’ they choose to deny having any suicidal thoughts. Alternately, some Veterans tell us what they think we want to hear rather than how they actually feel because they want to be perceived as doing well in therapy or on a test (yes, scholastic assessment carries over for a long time!)”* [ID0041].

Alternative ideas were offered to explain response distortion, with one provider sharing, *“Some patients who are therapy dependent react negatively to the positive outcomes. Some patients learn to answer negatively because they think that will keep them in therapy longer or look better for disability claims”* [ID0043]. Leaning into the concerns about disability benefits, one provider responded, *“Possibly some Veterans will over-report out of concern that their service connection could be impacted (though I think this is a minority in actuality)”* [ID0007.] Another stated, *“some Veterans are hesitant to answer honestly for fear of reduction in disability/benefits time”* [ID0037].

#### Structural barriers to provider engagement

Providers cited structural barriers to using tMBC, including concerns over asynchronous collection of time sensitive data (i.e., suicidality screening), functional and technical constraints of software used to support tMBC, and overall time constraints.

Three instances were offered as examples of problems that arise due to asynchronous MBC. The first concerned timing and coordination with the EHR scheduling software. Providers noted that confounds arise when a Veteran completes their assessments ahead of time but then cancels or misses the appointment, as their assessment data then lacks an associated clinical visit and cannot be uploaded. The next concern about asynchronous assessment also related to cancellation but referenced the burden on the provider to keep track of what forms were sent out if a Veteran cancels and reschedules an appointment. Finally, results from an asynchronous assessment of symptoms may trigger the need for emergent follow up, which is problematic when follow up contact with the patient is unsuccessful. Clinician responses to a positive suicide screen gathered during asynchronous assessment may need to be dynamic, as the symptom measures are not always sensitive to acute versus chronic suicidal ideation, both of which can occur in Veterans accessing care. Providers conveyed the need to navigate legal and ethical clinical practice standards when working with individuals who report suicidality.

Various functional and technical constraints were identified by participating providers. The library of assessments offered within BHL Touch was noted by several providers to lack assessments that are needed for some patients. Some providers still utilized assessments that were not in BHL Touch, with one respondent stating, *“I have been finding other ways to readily integrate other*,* more relevant measures into my practice that are not a part of the VA catalogue. But this does create issues with time in session*,* especially for Veterans who are being seen virtually and cannot fill out the measure in session”* [ID0033].

One provider described workflow inefficiencies when standardized assessments are not available through BHL Touch, noting that alternate methods (e.g., email or patient portal) often reduce response rates and require using valuable session time to complete measures that could otherwise be done independently.

There are times when something is not on BHL touch, and in those cases, I have to e-mail or MHV [My HealthEVet, patient portal] the info (which then I typically don’t get anything back) or I have to complete the assessment verbally (which takes session time I prefer to spend doing something non-standardized). For example, I am a provider in an OCD study which asks for the Y-BOCS to be filled out 1x/2 sessions. I have to e-mail rather than have a text sent, and the Veteran tends to fill it out in session with me rather than getting it done ahead of time. When I use BHL touch for the same Veteran to complete a PHQ and PCL, they typically get it done outside of session [ID0006].

Technical issues reported were related to software malfunctions, accessibility (if a Veteran’s device does not allow them to see the provider or screen during a video visit), and the impact of technical failures on the provider and patient relationship. One provider expressed, *“When it doesn’t work*,* it can be very frustrating (this can be especially important during an intake assessment when there may be high suspicion or nervousness coming into the appt)”* [ID0006].

Concern about technological proficiency among Veterans was reported as a barrier to MBC. Some providers reported a lack of confidence in their Veteran-patients’ ability to access and navigate the technology used by VHA in MBC (i.e., BHL Touch). On the other hand, one provider spoke about how providers need to be able to respond to Veteran’s preferences, “i*n this day and age the patients dictate the level of technology they are comfortable with yet as providers we need to be familiar with all options”* [ID0036].

#### Time as a barrier

Issues around time as a barrier to uptake of MBC were reported by 28% (*n* = 13) of respondents. When queried about the potential drawbacks of MBC, some providers simply responded with one word, “*time*”. Others shared more nuanced perspectives, noting that the time required to complete surveys during sessions and to review the data for discussion and treatment planning impacted adoption. One provider explained, “*the biggest problem I have is time required if the assessments are completed in session. BHL is a great tool*,* but Veteran compliance varies”* [ID0002]. Time as a barrier crossed over with reported Veteran engagement issues, with one provider noting, “*it does take session time and often patients aren’t as interested in that as other things*,* so it feels like a struggle to make them do MBC stuff”* [ID0039].

Another provider suggested that time constraints are related to reviewing and planning, stating a barrier to adoption is *“needing the time to review the assessments. Needing the time to adjust the course of overall treatment when assessments suggest so”* [ID0024].

The concern for time was not universal, with one provider stating *“I don’t know of any drawbacks. Time is really not an issue when you get used to it” [ID0019].* Despite that provider’s enthusiasm, the sentiment was not universally shared and participating providers offered important insights into the drawbacks of MBC that impact its usability in clinical care.

#### Measure validity and therapeutic value

Provider reports on barriers to fidelity to MBC as an evidence-based practice included not always understanding the therapeutic value of MBC, the predictive validity of specific measures and their sensitivity to change over the course of treatment, and how to respond clinically when Veteran’s scores did not indicate clinical improvement. For example, providers relayed their perception that scores are not necessarily always accurate, that “*measures don’t always accurately capture change*” [ID0020]. Some providers noted that the since the assessments are symptom-focused, they do not assess treatment goals that are non-symptom related, such as interpersonal or functional goals, with one provider sharing, “[MBC] *does not always capture important qualitative elements of change (e.g.*,* sense of confidence*,* improvement in relationships*,* sense of social connectedness*,* movement toward personal life goals like applying for jobs or school). Some therapy goals may be more interpersonal and less symptom focused*,* or some symptoms may be expected to demonstrate less overt change that can be tracked easily*,* such as personality disorders or SMI [serious mental illness]”* [ID0041].

One provider indicated that sometimes assessment scores do not match the lived experiences of Veterans, stating *“although the scores can be helpful on eliciting more discussion about certain symptoms I don’t think they are necessarily very accurate for various reasons; i.e. the Veteran’s perception of their score upon further discussion does not always match what one would assume if just looking at numbers”* [ID0026].

Beyond concerns about item or total score validity, providers shared uncertainty regarding the optimal frequency of assessment administration (e.g., how frequently the assessments are sent out) so as to not unduly burden patients and to detect meaningful symptom severity changes and inform treatment planning.

#### Stagnation or decline

Providers expressed concerns about the potential for challenges navigating clinical care, for the clinician or the Veteran, when a Veteran’s symptom scores lack evidence of clinical improvement. One provider stated that when Veterans’ assessment scores do not improve over the course of treatment, this can have a demoralizing effect for Veterans and can negatively impact clinicians, sharing *“Demoralization if their scores don’t change. If there are structural reasons the treatment will need to end (i.e.*,* come to end of protocol*,* etc.) and the scores are not improved*,* it makes it hard for the clinician to handle that clinically”* [ID0039]. In addition, this provider noted that it is challenging to utilize assessment results on a systems level due to other clinicians’ lack of utilization and this can decrease their motivation to use MBC, stating *“My interest in it at a program/team/clinic level is hard because most people don’t use it. So it’s hard to get the data I want from it. And then over time*,* it impacts my own individual motivation to use it”* [ID0039].

Some providers shared concern that MBC scores are viewed as the main indicator of patient outcomes and that their clinical performance would be evaluated based on their patients’ MBC results. One provider reported, “*I have heard providers express concern that their clinical performance/efficacy will be judged according to the Veterans’ MBC results”* [ID0007].

Another shared, *“They are not the end all be all. There are a variety of reasons why someone may score a certain way and that needs to be discussed and taken into account. At times it feels like VA places too much emphasis on MBC being the main driving indicator of therapy success”* [ID0011].

### Educational needs

Providers requested training in psychometric properties of assessments to inform selection and interpretation, how to talk to Veterans about the Collect, Share, and Act components of MBC, and workflow and integration into their own practice. They asked for small-group or step-by-step training, workflow guidelines, and a full assessment directory. Respondents spoke about the need for comprehensive training in MBC, with one provider sharing her belief that when MBC is used by providers who are poorly trained, it leads to poor patient experience.

#### Assessments selection and interpretation

Two overarching educational needs around assessment selection and interpretation were identified: (1) awareness and understanding of assessments and psychometric properties to inform selection and interpretation and (2) knowing which assessments best capture symptom burden related to specific diagnoses.

Providers broadly indicated a need for a better understanding of what is available, with one provider suggesting the desire for a “directory of measures”. One provider asked for a “*resource including what other providers use when treating a specific condition and why”* [ID0001]. Another provider spoke to the gap in available assessments, reporting, *“I am having problems finding a good assessment tool to use for anger management. Most published measures regarding anger are ‘trait’ measures rather than a ‘state’ measure and not that useful for repeat assessment”* [ID0002].

Beyond awareness of what is available, providers shared that to feel more confident in using MBC, they need to better understand psychometric properties of the assessments available on BHL Touch and what constitutes clinically meaningful change in scores. Several providers emphasized the need for additional guidance on how to interpret assessment results and apply them meaningfully in treatment, noting that further training would help them use these tools more effectively. Providers specifically expressed interest in learning how they can translate scores into treatment planning. One provider noted that a barrier to adherence was “*Not fully understanding the meaning gained from some assessments*” [ID0024]. Others described what would help them, sharing they would like *“Education on the specific meaning/interpretation of the assessments and how they could inform the treatment and give meaning to Veterans for the major assessments*” [ID0024], and “*Training to better help the providers to understand the results of the tools and how to utilize them effectively in treatment”* [ID0042]. One provider reported a detailed and insightful educational need related to understanding the minimal important difference for the measures, stating that education would be helpful on

What are statistically significant changes in total score for some of the more common measures (PHQ9, GAD, PCL, CESD). I.e. I think I’ve heard 5 pts for phq9 but I am not sure so this is just a fake example: Say you are working with a pt with a depression goal and although your pt’s total score improvement did not trend at/below minimal depression, but say they had a (greater than) or = 5 pt reduction in total score from 18 to 12 and this is deemed statistically significant for this measure meaning that they likely saw a meaningful change and this is still considered a successful episode of care…[ID0045].

A need for nuanced education around selection and interpretation of assessments that best measure symptoms associated with particular mental health conditions or patient circumstances was reported. Personality disorders were identified as challenging diagnoses for utilizing MBC, as some providers indicated it is difficult to find brief assessments that sensitively assess personality disorder symptoms.

#### Provider-delivered psychoeducation

Providers consistently asked for education on how to discuss MBC with Veterans. Specifically, providers want to know how to explain the meanings and interpretations of the assessments, how to inform Veterans of how their results will impact their treatment”, how to discuss symptom changes (or lack thereof), and how to make discussing MBC more relatable to the Veteran patient. One provider responded, “*How to show results of assessments over time over VVC*,* just some discussion about helpful ways to talk about use of measurements with Veterans*,* and education about how to best use some of the measures (info about the purpose*,* data about reliability/validity)”* [ID0032].

Some providers sought nuanced education on specific topics, such as how to discuss scores that do not align with patients’ narrative reports or how to address changes over time, effective implementation when caring for patients presenting with therapy blocking behaviors, or who may be experiencing higher levels of distress in an outpatient setting. One provider noted the need for education related to caring for patients with particular diagnoses, sharing “*MBC troubleshooting for certain presenting concerns -- for example in the DBT* [Dialectical Behavioral Therapy] *team*,* where treatment can last for a year*,* we don’t have great options for MBC for patients with BPD* [Borderline Personality Disorder]*- we have tried a range of them like the DERS* [Difficulty in Emotion Regulation Scale], *BSL-23* [Borderline Symptom List 23]*- none of them capture what we want to track for individual patients and other measures are not available on MHA* [Mental Health Assistant] */BHL/VHA system. Other personality disorders as well - not a lot of brief options for tracking change in the first place (generally extremely lengthy!)*,* and PDs* [Personality Disorders] *may not be expected to show significant changes in discrete symptom count”* [ID0040].

#### Workflow and integration into clinical practice

Providers reported broad educational needs related to workflow, varying from broad requests of “*anything to streamline the workflow*” [ID0007] to a “*step-by-step process*” [ID0028]. Education needs around technology varied. Some providers reported a need for fundamental training on the use of BHL Touch software, such as how to log in, send the surveys to the Veterans, and integrate the assessments into the EHR. One provider requested education on how to work with BHL to add assessments to the library, stating “*Getting more measures built into the programs. I have a niche measure for my ADHD patients that others might find very insightful*,* but it’s not on any of the MBC programs. I have to deliver it via WebEx Poll any time I want to use it*,* which is very cumbersome and time consuming*,* but worth it”* [ID0041].

Another issue providers reported needing help navigating is more than one licensed independent practitioner (LIP) may be using MBC with the same patient, with one provider asking for information on “*which LIPs should be administering them*” [ID0036], speaking to the nuances of engaging in MBC in a multidisciplinary setting.

## Discussion

This qualitative inquiry to assess provider perceptions of MBC to inform development of a digital MBC workflow in a tele-health setting revealed nuanced views of its clinical utility, barriers to adoption, and areas for provider training—directly addressing the initial research aim. Findings demonstrate the intersection of digital health implementation and behavioral healthcare delivery, highlight the nuanced factors influencing tMBC adoption in VHA telehealth settings, and underscore how workflow integration and user-centered design remain critical determinants for successful adoption of digital tools in telehealth environments.

In descriptions of the benefits of MBC, providers recognized its potential to enhance symptom awareness and monitoring, support treatment planning, and facilitate patient engagement in therapy. Regarding barriers to uptake of tMBC, they shared persistent barriers that limited routine use such as time constraints, inconsistent Veteran participation, functionality of digital tools, and challenges in interpreting and applying results. Educational needs reflected barriers identified, with requests for training centered around psychometric proprieties of measures, effective communication strategies with Veterans, and guidance on integrating digital tools into workflows.

Consistent with prior implementation work [32–34], providers emphasized a need for structured guidance on communicating results to Veterans—a gap that may be addressed through MBC-specific psychoeducational tools. Providers requested language that could specifically be used in clinical encounters to help Veterans understand the utility and value of MBC. Based on this feedback, it was determined that providers need training and ongoing clinical support on how to provide psychoeducation on MBC that effectively educates the Veteran patients on all components of this clinical tool, ranging from risks and benefits to sharing results that provide insight into the illness and meaningfully impact treatment decisions. Psychoeducation is a vital element of widely used psychotherapeutic models like Cognitive Behavioral Therapy [35, 36], Dialectal Behavioral Therapy [37], Acceptance and Commitment Therapy [38], and Psychodynamic Psychotherapy [39]. Mental health providers who use these models during therapeutic interventions have been taught how to provide psychoeducation in the context of that model through manuals, training, certification courses, etc. However, there is a gap in the literature regarding how to provide psychoeducation specific to the MBC model [2, 40–42]. Similar challenges have been reported in other disciplines- such as physical therapy [43], occupational therapy, and physiotherapy [44]-that also integrate assessment and measurement into routine clinical care.

Concerns about how to manage suicidality disclosures highlight the need for standardized communication protocols and informed consent procedures within digital MBC workflows along with ongoing clinical support for individual cases. Across the United States, post-screen responses to a positive suicide screen vary by setting, resources, and local policy, in part because national accrediting [45] and quality frameworks [46–48] emphasize that organizations should have written procedures for screening, assessment, and follow-up, but do not mandate a single uniform operational pathway. For example, some systems pair screening with brief interventions and proactive follow-up contacts (e.g., safety planning and follow-up phone calls) that have demonstrated benefit in reducing subsequent suicidal behavior in emergency care settings, whereas other settings rely more heavily on urgent escalation pathways (e.g., same-day in-person assessment, emergency services involvement, or hospitalization) depending on acuity and available staffing. Across VHA, providers using tMBC work on interdisciplinary teams, with each discipline having their own best practices for responding to positive suicide screens whilst working together to ensure overall best care and safety of a Veteran. In discussing the measures selected for a patient, providers have an opportunity to educate the Veteran about the implications of a positive responses on suicide-related items.

Successful adoption of tMBC must include education and an emphasis on MBC’s central tenant that clinical symptom rating and other assessment scales are tools to be used in concert with history-taking and clinical judgment, which is always paramount for an effective treatment approach. Furthermore, selection of appropriate assessment tools requires knowledge of the psychometric properties of the individual tools in the context of clinical assessment and clinical judgement. This can be an important factor when providers are considering measures with the potential to capture self-report bias, such as over or under-reporting [49], which could confound effective treatment. Whether an assessment score stagnates, worsens, or improves, clinicians need reassurance that there is always a need for interpretation of the measurement data in the context of the overarching clinical assessment, including the indications for further inquiry, the use of collateral informants, consultation with other colleagues, and consideration of the appropriateness or validity of the scale/measure for that specific patient’s condition. For example, changes in sleep patterns and the absence of depressive phenomena on the PHQ-9 could reflect cycling from a depressed to a manic state or cognitive decline could impact the ability of a patient to respond using a self-report scale.

### Integration of clinical, technical, and administrative requirements to inform the implementation strategy

The findings from this pre-implementation evaluation informed refinement of a multi-component implementation strategy that addresses interdependent clinical, technical, and administrative requirements necessary for effectively delivered tMBC. Rather than viewing provider-identified benefits, drawbacks, and educational needs as discrete findings, we synthesized them across these domains to develop a cohesive implementation bundle aligned with VHA’s “Collect, Share, Act” framework [12] and existing MBC implementation literature [2, 5, 40]. In 2025, the American Psychological Association [50] released formal guidelines on MBC. Although this project preceded the publication of those guidelines, its aims were closely aligned—particularly the emphasis on monitoring treatment goals beyond symptom reduction. The primary goal of this project was to strengthen providers’ understanding of MBC as a therapeutic tool rather than solely a screening mechanism. To support this goal, we educated psychotherapy providers on the broader clinical utility of standardized measures and to support their learning, developed and distributing a binder containing a hard copy of all assessments available within BHL Touch.

#### Clinical requirements

Providers identified clinical value in MBC for enhancing symptom awareness and guiding treatment planning. At the same time, concerns emerged regarding interpretation of scores, response distortion, management of suicidality disclosures, and therapeutic responses to stagnant or worsening scores. In response, the implementation strategy incorporated structured provider training modules focused on psychometric literacy, clinical integration of scores within broader case formulation, and communication tips to support collaborative interpretation of results with Veterans. These components align with calls in the literature for workforce development as a central mechanism of sustainable tMBC implementation [2, 40, 41] and reinforce that MBC improves outcomes only when results are actively shared and discussed [6, 10]. By embedding education about psychometrics and clinically significant differences within routine workflow, the strategy operationalizes the “Share” and “Act” components rather than relying solely on “Collect” (i.e., assessment administration).

#### Technical requirements

Providers emphasized that technological functionality and workflow efficiency as adoption determinants. Barriers such as asynchronous suicide alerts, limited assessment libraries, EHR integration challenges, and time burden reflect known informatics constraints in digital MBC systems [18, 20, 23]. Accordingly, the QI project’s implementation strategy prioritized optimization of electronic assessment capture through BHL Touch and clarification of processes for managing time-sensitive responses. Workflow mapping exercises during the Design phase reduced redundancies and clarified responsibilities for sending, reviewing, and documenting assessments. These adaptations are consistent with digital measurement feedback system research demonstrating that usability and integration into clinical workflow are critical predictors of sustained use [18] and that technological capability alone is insufficient without human-centered design [25–27].

#### Administrative requirements

Providers’ concerns regarding performance evaluation, role delineation among LIPs, and documentation burden highlight the administrative context in which tMBC is implemented. Thus, the QI project’s implementation strategy included leadership engagement, discussion of roles in assessment administration in multidisciplinary settings, and education on documentation practices that align with EHR capabilities. Framing MBC as a clinical enhancement tool rather than a performance surveillance mechanism was particularly important in addressing provider apprehension and promoting psychological safety.

Our findings are consistent with prior literature demonstrating that tMBC implementation succeeds when it incorporates training, leadership support, workflow redesign, and digital infrastructure as coordinated components rather than standalone initiatives [5, 18, 40]. Importantly, the clinical, technical, and administrative domains are interdependent. For example, technical optimization reduces time burden (administrative), which in turn enhances provider willingness to engage in collaborative interpretation (clinical). Likewise, clear administrative policies regarding suicide response protocols enable clinicians to confidently use digital assessments without fear of unmanaged risk. By grounding the QI project’s implementation strategy development in provider-identified needs during the Discover phase, this project advanced from best-practice recommendations toward contextually tailored standard practice, echoing recent calls to shift tMBC from aspirational to operational within health systems [11].

Collectively, this pre-implementation work demonstrates that addressing clinical competence, technological usability, and administrative alignment in tandem is essential for realizing the full promise of digitally delivered MBC in rural tele-mental health settings. Future evaluation phases can examine whether this integrated implementation strategy improves adoption, fidelity to “Collect, Share, Act,” and ultimately Veteran-level outcomes.

### Implications

This study contributes to the growing field of digital health by demonstrating how technology-enabled measurement and feedback systems can enhance the reach and quality of mental health services. VHA’s infrastructure and data capacity make it a powerful setting for MBC growth, yet the success of MBC depends on equipping providers with the knowledge and confidence to select, use, and apply assessment tools meaningfully. Providers’ experiences underscore how digital tools, such as BHL Touch and VHA Video Connect, serve as digital health enablers that improve coordination, data flow, and patient engagement—core dimensions of digital healthcare transformation. Findings from this inquiry are specific to the utilization of BHL Touch, however, it could apply to the utilization of other software programs such as Mental Health Assistant or eScreening. Embedding tMBC within digital health infrastructures supports the VHA’s mission to expand access to high-quality, data-driven care, particularly for rural and underserved Veterans.

#### Future work

Future work could explore various components of implementation of the Collect, Share, Act model including how artificial intelligence platforms may address provider workflow issues; how using mobile or electronic health applications (i.e. VA’s patient portal My Health*e*Vet) may facilitate patients’ engagement with data collected in and between sessions; and how to broaden the adoption of functional assessments used in concert with symptom assessments.

Our approach tailored the training modules to the psychotherapists within a Mental Health Service Line. However, future work could explore the impact of tailored, discipline-specific training modules addressing psychometrics of self-report assessment tools, digital workflow, and communication strategies to enhance provider confidence and uptake.

### Strengths and limitations

This study has several strengths and limitations that should be considered when interpreting its findings. A key strength lies in its timely discovery of provider perspectives to inform the design and build phases in a QI initiative within VHA. By capturing provider insights early, the data directly informed subsequent design and workflow adaptations, exemplifying a learning health system approach [1].

The sample included two mental health disciplines serving rural and highly rural Veterans across a large geographic area, offering a rich view of barriers and facilitators to implementing MBC in virtual settings. The use of inductive thematic analysis and multiple coders enhanced analytic rigor and credibility.

Nevertheless, the study has limitations. Qualitative data were collected through open-ended survey responses rather than in-depth interviews or focus groups. Although this approach facilitated participation from a geographically dispersed provider group, it limited opportunities for follow-up probing and contextual clarification. Providers’ responses may be subject to self-selection and social desirability bias, as providers who were more engaged or favorable toward MBC may have been more likely to participate or to emphasize positive aspects of implementation. The survey was developed de novo and was not pilot-tested or validated, which may influence content comprehensiveness and construct coverage. Finally, the analysis reflects perspectives from providers within one VHA Mental Health Service Line and may not be generalizable to all VHA settings or non-VHA health systems.

As this work represents the Discover phase of a quality improvement project, findings capture anticipated rather than observed implementation challenges and outcomes.

Despite these limitations, the results provide valuable early insights into provider readiness, perceived barriers, and educational needs that directly informed subsequent phases of MBC implementation within virtual mental health care delivery.

Further exploration of Veteran experiences with MBC/tMBC is warranted to better understand how adoption and fidelity at a provider level impact Veteran care experience and outcomes. As part of this QI project, qualitative data were collected from a sample of Veterans who received care from providers working in the Mental Health Service Line (manuscript forthcoming).

## Conclusion

The results detailed in this manuscript informed the development of an implementation strategy that identified core clinical, technical, and administrative requirements necessary for effective, digitally delivered MBC, while allowing for variability across settings and underlying information technology platforms. For this QI project, providers’ nuanced perceptions of benefits, drawbacks, and educational needs was paramount to the Design, Build, and Test phases of the project.

This project underscores that while VHA is well-positioned to lead in MBC implementation, adoption is not automatic. A structured, provider-informed, and Veteran-sensitive approach is essential. By educating providers on psychometric properties of assessments and integrating digital tools like BHL Touch with workflows, training, and communication strategies, MBC can become a routine, meaningful part of digital mental health care delivery in the VHA and beyond. As digital health ecosystems continue to evolve, integrating MBC into telehealth delivery offers a scalable pathway to improving mental health care access, personalization, and continuity for diverse populations.

## Supplementary Information

Below is the link to the electronic supplementary material.

Supplementary Material 1

## Data Availability

The qualitative data generated and analyzed during the current study is not publicly available in accordance with VA policy governing research data collected within the VA system. Although the study did not involve Veteran patients, the data consists of surveys completed by VA mental health providers and include potentially sensitive information about VA clinical practices, organizational processes, and internal operations. Per VA policy on research data management and access, such data may not be shared outside the VA without explicit authorization and a formal Data Use Agreement approved by the local VA Research & Development Committee.

## Declarations

### Artificial intelligence

Portions of this manuscript were edited with the assistance of an artificial intelligence (AI) tool, ChatGPT-5, OpenAI. Outputs underwent rigorous human review to ensure accuracy, clarity, and appropriateness for scholarly publication. Consistent with International Committee of Medical Journal Editors recommendations [51], the authors maintained full responsibility for the content and interpretation of the manuscript. The use of AI was limited to language refinement and did not involve interpretation of study data.

## Abbreviations

BASIS-24: Behavior and Symptom Identification Scale–24
BHL Touch: Behavioral Health Lab Touch
BPD: Borderline Personality Disorder
CBOC: Community-Based Outpatient Clinic
CES-D: Center for Epidemiologic Studies Depression Scale
DBT: Dialectical Behavioral Therapy
DERS: Difficulties in Emotion Regulation Scale
HER: Electronic Health Record
LIP: Licensed Independent Practitioner
MBC: Measurement-Based Care
MHV: My HealtheVet
PCL: PTSD Checklist (Version 5)
PHQ-9: Patient Health Questionnaire-9
PTSD: Post-Traumatic Stress Disorder
QI: Quality Improvement
QUERI: Quality Enhancement Research Initiative
SAS: Statistical Analysis System
SMI: Serious Mental Illness
tMBC: Telehealth-delivered Measurement Based Care
VAMC: Veterans Affairs Medical Center
VHA: Veterans Health Administration
VVC: VA Video Connect
