# Supplementary material for: Implementing measurement-based care in virtual mental health services for rural veterans: provider insights from a pre-implementation evaluation
Source: BMC Health Serv Res. 2026 Apr 3;26:694. doi: 10.1186/s12913-026-14490-6 (PMC13173779; doi:10.1186/s12913-026-14490-6)
Supplement: Supplementary file 1 — Supplementary Material 1 [file 12913_2026_14490_MOESM1_ESM.docx]

**MBC Provider Baseline Survey**

**Q1 Measurement-Based Care**

Measurement-Based Care (MBC) refers to the standard assessment of patient-reported outcome throughout the course of treatment. Examples of commonly used assessments in MBC are the 9-Item Patient Health Questionnaire, the PTSD Checklist -5, or the Insomnia Severity Index. In MBC, the provider and patient review responses to these assessments to help guide decisions about care.

Please indicate the extent to which you agree with the following statements about MBC. Please respond even if you don’t currently use MBC widely in your practice.

|  | Strongly Disagree (1) | Disagree (2) | Neither Agree nor Disagree (3) | Agree (4) | Strongly Agree (5) |
| --- | --- | --- | --- | --- | --- |
| MBC helps patients and providers to better identify the goals of treatment. (1) |  |  |  |  |  |
| Patients find value in completing standard outcome assessments and discussing them during visits. (2) |  |  |  |  |  |
| MBC helps to identify patient symptoms or problems providers may not be aware of. (3) |  |  |  |  |  |
| MBC helps patients better understand their mental health conditions. (4) |  |  |  |  |  |
| MBC makes it easier to assess the effectiveness of treatment. (5) |  |  |  |  |  |
| MBC improves how patients and providers make decisions about treatment. (6) |  |  |  |  |  |
| MBC enhances the therapeutic alliance between patients and providers. (7) |  |  |  |  |  |

|  | Strongly Disagree (1) | Disagree (2) | Neither Agree nor Disagree (3) | Agree (4) | Strongly Agree (5) |
| --- | --- | --- | --- | --- | --- |
| It would be easy for providers to use eHealth tools, such as BHL Touch or MH Checkup, to administer MBC assessments. (1) |  |  |  |  |  |
| Patients would find it easy to use a secure electronic link, via text or email, to complete MBC assessments. (2) |  |  |  |  |  |
| Providers would have enough time in their clinical practice to conduct MBC effectively. (3) |  |  |  |  |  |
| It would be easy to implement MBC with patients seen via telehealth. (4) |  |  |  |  |  |
| It would be easy to review MBC results with patients during a telehealth visit. (5) |  |  |  |  |  |
| Patients with more severe mental health symptoms would be able to engage effectively in MBC. (6) |  |  |  |  |  |

Have you used MBC when conducting a formal evidence-based practice with your patients such as therapies to improve sleep, Cognitive Processing Therapy or Prolonged Exposure Therapy?

- Yes (1)
- No (2)

Have you used MBC with your patients who are in pharmacotherapy, supportive therapy, or who are not currently enrolled in a formal evidence-based practice described above?

- Yes (1)
- No (2)

With what percentage of your patients do you use measurement-based care?

|  | 0 | 10 | 20 | 30 | 40 | 50 | 60 | 70 | 80 | 90 | 100 |
| --- | --- | --- | --- | --- | --- | --- | --- | --- | --- | --- | --- |

In your opinion, what are the major potential benefits of MBC?

In your opinion, what are the major potential drawbacks of MBC?

What would be helpful to you in terms of education about MBC? For example, providers may want more information about specific software programs used, how to discuss results with your patient or anything else related to this topic?

Please indicate your training background:

- Psychologist (1)
- Physicians Assistant (2)
- Licensed Clinical Social Worker (3)
- Mental Health Physician (M.D., D.O.) (4)
- Primary Care Physician (5)
- Medical Services Assistant (6)
- Behavioral Health Nurse (7)
- Licensed Nurse Practitioner (8)
- Licensed Marriage and Family Therapist (9)
- Licensed Professional Counselor (10)
- Pharmacist (11)
- Other, Please specify (12) __________________________________________________
- Prefer not to answer (13)

Please indicate your age:

- 25-39 (1)
- 40-59 (2)
- 60+ (3)
- Prefer not to answer (4)
